# Editorial on Special Collection in *Contact*: VPS13 and Bridge-Like Lipid Transfer Proteins: A New Mode of Intracellular Continuity

**DOI:** 10.1177/25152564241238538

**Published:** 2024-03-15

**Authors:** Tim P Levine, Liz Conibear

**Affiliations:** UCL Institute of Ophthalmology, London, UK; Centre for Molecular Medicine and Therapeutics, British Columbia Children's Hospital Research Institute, University of British Columbia, Vancouver, Canada

There has been an explosion of interest in vacuolar protein sorting 13 (VPS13) and related proteins, with a 10-fold increase in the rate of publication of papers from 10 per year before 2015 to 100 now. The path that led to this started with developments that strongly implicated the yeast Vps13 protein in lipid transfer (Lang et al., 2015). The landmark discovery came in 2018, when it was shown that these large proteins transfer lipids (Kumar et al., 2018). Soon after came the realization that Vps13 and Atg2 are structurally related, marking them as founding members of a larger protein family (Osawa et al., 2019; Valverde et al., 2019). Another key development here, and everywhere else, was when AlphaFold allowed us to see the bridge-forming structures of the entire family (10 in humans, seven in yeast, and six in flowering plants), many of which can bridge gaps between organelles (15–30 nm) (Jumper et al., 2021). To celebrate these advances, this journal put together a collection of new work on VPS13 and other bridge-like transfer proteins, which includes six original research articles and four reviews.

Starting with original research (see Table 1), Amos et al. showed that VPS13A interacts with XK, its known partner at the plasma membrane, only in cells at the right stage of differentiation, explaining why this was missed using standard approaches (Amos et al., 2023). Levine showed how domains, particularly the repeating beta-groove (RBG) domains characteristic of bridge-like lipid transfer proteins, are distributed across eukaryotes (Levine, 2022). Leterme et al. used bioinformatics to show the complex evolution of VPS13 in photosynthetic organisms, producing the groundwork for functional studies (Leterme et al., 2023). Du et al. showed that VPS13B, previously linked to the Golgi apparatus, has a positive role in promoting Golgi-LD contact, a cellular phenomenon hardly reported on before (Du et al., 2023). Chen et al. showed that both VPS13A and VPS13C, two proteins in this group that can target lipid droplets, are functionally linked to this compartment, being required for normal lipid accumulation (Chen et al., 2022). Lei et al. showed that in addition to the known role of ATG2 in supplying lipids to the growing autophagosome, Vps13 in yeast contributes at the late stages, with some selectivity for the type of autophagic cargo (Lei et al., 2022).

**Table 1.** Articles in Special Collection on VPS13 and Bridge-Like Lipid Transfer Proteins.

Lead Author	Topic	
	Original Research	Reference
Pietro De Camilli	VPS13A interaction with XK	Amos et al. (2023)
Tim Levine	Structural elements in bridge-like LTP superfamily	Levine (2022)
Morgane Michaud	Phylogeny of VPS13 in plants	Leterme et al. (2023)
Weike Ji and Juan Xiong	VPS13B at Golgi-lipid droplet contacts	Du et al. (2023)
James Olzmann and Susan Ferro-Novick	VPS13A/VPS13C and lipid droplet abundance	Chen et al. (2022)
Dan Klionsky	Vps13 in autophagy in yeast	Lei et al. (2022)
	Reviews	
Ruth Walker	Neuroacanthocytosis syndromes: clinical perspective	Walker et al. (2023)
Natasha Ruiz	Bridge-like LTP antecedents in bacteria	Kumar and Ruiz (2023)
Dengke Ma	Bridge-like LTPs in *C. elegans*	Pandey et al. (2023)
Fulvio Reggiori	Phagophore-ER contacts	Vargas Duarte and Reggiori (2023)

Among the reviews, Walker et al. described the “core neuroacanthocytosis syndromes,” which indicate a shared pathway for VPS13A and XK, so that diseases of each, previously called chorea-acanthocytosis and McLeod syndrome, respectively, are currently recognized as highly similar (Walker et al., 2023). Kumar et al. described the AsmA-like protein family in Gram-negative bacteria. These descendants of the likely prokaryotic ancestor of RBG proteins are one of two large protein assemblies recently shown to transfer phospholipids from the inner membrane to the outer membrane (Kumar and Ruiz, 2023). Pandey et al. described the superfamily of bridge-like lipid transfer proteins in *C. elegans*, in particular focusing on their own work on LPD-3, the ortholog of BLTP1/Tweek/Fmp27, which provides a useful model for the BLTP1-associated hereditary neurodevelopmental disorder Alkuraya-Kučinskas syndrome (Pandey et al., 2023). Finally, Vargas Duarte and Reggiori described the key AuTophaGy (ATG) proteins for lipid traffic to growing autophagosome membranes: the RBG protein ATG2 and its partners on autophagosomes ATG18/WIPI4 and ATG9, which act as an adaptor and lipid scramblase, respectively, (Vargas Duarte and Reggiori, 2023).

The discovery that these large proteins are direct conduits for lipid flow between organelles has upended our ideas about eukaryotic lipid transport. The papers in this special collection have outlined new roles for bridge-like transfer proteins and emphasized general principles. They also highlighted fundamental open questions (e.g., What powers lipid traffic and what regulates it?) which will no doubt prove fascinating areas for future research.

## References

[bibr1-25152564241238538] Amos C Xu P De Camilli P (2023). Erythroid differentiation dependent interaction of VPS13A with XK at the plasma membrane of K562 cells. Contact (Thousand Oaks) 6, 25152564231215133. doi: 10.1177/25152564231215133 38144430 PMC10748539

[bibr2-25152564241238538] Chen S Roberts MA Chen CY Markmiller S Wei HG Yeo GW Granneman JG Olzmann JA Ferro-Novick S (2022). VPS13A and VPS13C influence lipid droplet abundance. Contact (Thousand Oaks) 5, 25152564221125613. doi: 10.1177/25152564221125613 36147729 PMC9491623

[bibr3-25152564241238538] Du Y Hu X Chang W Deng L Ji WK Xiong J (2023). A possible role of VPS13B in the formation of Golgi-lipid droplet contacts associating with the ER. Contact (Thousand Oaks) 6, 25152564231195718. doi: 10.1177/25152564231195718 38090145 PMC10714374

[bibr4-25152564241238538] Jumper J Evans R Pritzel A Green T Figurnov M Ronneberger O Tunyasuvunakool K Bates R Zidek A Potapenko A , et al. (2021). Highly accurate protein structure prediction with AlphaFold. Nature 596, 583–589. doi: 10.1038/s41586-021-03819-2 34265844 PMC8371605

[bibr5-25152564241238538] Kumar N Leonzino M Hancock-Cerutti W Horenkamp FA Li P Lees JA Wheeler H Reinisch KM De Camilli P (2018). VPS13A and VPS13C are lipid transport proteins differentially localized at ER contact sites. J Cell Biol 217, 3625–3639. doi: 10.1083/jcb.201807019 30093493 PMC6168267

[bibr6-25152564241238538] Kumar S Ruiz N (2023). Bacterial AsmA-like proteins: Bridging the gap in intermembrane phospholipid transport. Contact (Thousand Oaks) 6, 25152564231185931. doi: 10.1177/25152564231185931 37455811 PMC10345924

[bibr7-25152564241238538] Lang AB John Peter AT Walter P Kornmann B (2015). ER-mitochondrial junctions can be bypassed by dominant mutations in the endosomal protein Vps13. J Cell Biol 210, 883–890. doi: 10.1083/jcb.201502105 26370498 PMC4576869

[bibr8-25152564241238538] Lei Y Wen X Klionsky DJ (2022). Vps13 is required for efficient autophagy in *Saccharomyces cerevisiae*. Contact (Thousand Oaks) 5. doi: 10.1177/25152564221136388 PMC10162780 37151407

[bibr9-25152564241238538] Leterme S Bastien O Aiese Cigliano R Amato A Michaud M (2023). Phylogenetic and structural analyses of VPS13 proteins in archaeplastida reveal their complex evolutionary history in viridiplantae. Contact (Thousand Oaks) 6, 25152564231211976. doi: 10.1177/25152564231211976 38033810 PMC10683392

[bibr10-25152564241238538] Levine TP (2022). Sequence analysis and structural predictions of lipid transfer bridges in the repeating beta groove (RBG) superfamily reveal past and present domain variations affecting form, function and interactions of VPS13, ATG2, SHIP164, hobbit and tweek. Contact (Thousand Oaks) 5, 251525642211343. doi: 10.1177/25152564221134328 36571082 PMC7613979

[bibr11-25152564241238538] Osawa T Kotani T Kawaoka T Hirata E Suzuki K Nakatogawa H Ohsumi Y Noda NN (2019). Atg2 mediates direct lipid transfer between membranes for autophagosome formation. Nat Struct Mol Biol 26, 281–288. doi: 10.1038/s41594-019-0203-4 30911189

[bibr12-25152564241238538] Pandey T Zhang J Wang B Ma DK (2023). Bridge-like lipid transfer proteins (BLTPs) in *C. elegans*: From genetics to structures and functions. Contact (Thousand Oaks) 6, 25152564231186489. doi: 10.1177/25152564231186489 37455813 PMC10345909

[bibr13-25152564241238538] Valverde DP Yu S Boggavarapu V Kumar N Lees JA Walz T Reinisch KM Melia TJ (2019). ATG2 transports lipids to promote autophagosome biogenesis. J Cell Biol 218, 1787–1798. doi: 10.1083/jcb.201811139 30952800 PMC6548141

[bibr14-25152564241238538] Vargas Duarte P Reggiori F (2023). The organization and function of the phagophore-ER membrane contact sites. Contact (Thousand Oaks) 6, 25152564231183898. doi: 10.1177/25152564231183898 37465355 PMC10350784

[bibr15-25152564241238538] Walker RH Peikert K Jung HH Hermann A Danek A (2023). Neuroacanthocytosis syndromes: The clinical perspective. Contact (Thousand Oaks) 6, 25152564231210339. doi: 10.1177/25152564231210339 38090146 PMC10714877

